# Bead-based assays to simultaneously detect multiple human inherited blood disorders associated with malaria

**DOI:** 10.1186/s12936-019-2648-7

**Published:** 2019-01-21

**Authors:** Lynn Grignard, Catherine Mair, Jonathan Curry, Laleta Mahey, Guide J. H. Bastiaens, Alfred B. Tiono, Joseph Okebe, Sam A. Coulibaly, Bronner P. Gonçalves, Muna Affara, Alphonse Ouédraogo, Edith C. Bougouma, Guillaume S. Sanou, Issa Nébié, Kjerstin H. W. Lanke, Sodiomon B. Sirima, Umberto d’Alessandro, Taane G. Clark, Susana Campino, Teun Bousema, Chris Drakeley

**Affiliations:** 1 Department of Immunology and Infection, London School of Hygiene & Tropical Medicine, London, UK; 2 LGC Genomics, Hoddesdon, Hertfordshire UK; 3 Department of Medical Microbiology, Radboud University Medical Centre, Nijmegen, The Netherlands; 4 Department of Biomedical Sciences, Centre National de Recherche et de Formation sur le Paludisme, Ouagadougou, Burkina Faso; 5 Disease Control & Elimination Theme, Medical Research Council Unit at London School of Hygiene and Tropical Medicine, Fajara, The Gambia; 6 Department of Disease Control, London School of Hygiene and Tropical Medicine, London, UK; 7 Department of Pathogen Molecular Biology, London School of Hygiene and Tropical Medicine, London, UK; 8 Department of Infectious Disease Epidemiology, London School of Hygiene and Tropical Medicine, London, UK

**Keywords:** Malaria, Glucose-6-phosphate dehydrogenase deficiency, Haemoglobin S, Haemoglobin C, Multiplex detection

## Abstract

**Background:**

Glucose-6-phosphate dehydrogenase deficiency (G6PDd), haemoglobin C (HbC) and S (HbS) are inherited blood disorders (IBD) common in populations in malaria endemic areas. All are associated to some degree with protection against clinical malaria whilst additionally G6PDd is associated with haemolysis following treatment with 8-aminoquinolines. Measuring the prevalence of these inherited blood disorders in affected populations can improve understanding of disease epidemiology. Current methodologies in epidemiological studies commonly rely on individual target amplification and visualization; here a method is presented to simultaneously detect the polymorphisms and that can be expanded to include other single nucleotide polymorphisms (SNPs) of interest.

**Methods:**

Human DNA from whole blood samples was amplified in a novel, multiplex PCR reaction and extended with SNP-specific probes in an allele specific primer extension (ASPE) to simultaneously detect four epidemiologically important human markers including *G6PD* SNPs (G202A and A376G) and common haemoglobin mutations (HbS and HbC). The products were hybridized to magnetic beads and the median fluorescence intensity (MFI) was read on MAGPIX^®^ (Luminex corp.). Genotyping data was compared to phenotypical data generated by flow cytometry and to established genotyping methods.

**Results:**

Seventy-five samples from Burkina Faso (n = 75/78, 96.2%) and 58 samples from The Gambia (n = 58/61, 95.1%) had a *G6PD* and a *HBB* genotype successfully assigned by the bead-based assay. Flow cytometry data available for n = 61 samples further supported the concordance between % G6PD normal/deficient cells and genotype.

**Conclusions:**

The bead based assay compares well to alternative measures of genotyping and phenotyping for G6PD. The screening is high throughput, adaptable to inclusion of multiple targets of interest and easily standardized.

**Electronic supplementary material:**

The online version of this article (10.1186/s12936-019-2648-7) contains supplementary material, which is available to authorized users.

## Background

*Plasmodium* parasites have co-evolved with human hosts and exert a considerable evolutionary pressure on mutations that confer a degree of protection against malaria [1–5]. Examples of such mutations include polymorphisms in the X-linked glucose-6-phosphate dehydrogenase *(G6PD)* gene and in the *HBB* gene. G6PD is essential for the protection of cells against oxidative damage [6] and G6PD deficiency is common in malaria endemic areas [7] due to its association with protection of some manifestations of severe malaria [8]. The repertoire of G6PD deficiency molecular markers is extensive and displays considerable spatial heterogeneity [7, 9]. One of the most common *G6PD* variants in sub-Saharan Africa, the A− variant, requires two single nucleotide changes [10]. There are three common A− variants, an A376G substitution (present in all variants) may occur in combination with G202A, T968C or A542T substitutions. In The Gambia for example, the combinations of A376G/G202A, A376G/T968C and A376G/A542T are present in 1.8%, 2.1% and 1% of the population, respectively, while the A376G/G202A variant and A376G without additional *G6PD* mutations are commonly found in Burkina Faso [11]. *G6PD* A− homo-/hemizygosity is associated with a pronounced 88% loss in enzyme activity whilst *G6PD* A+ is associated with only a mild 15% loss in enzyme activity [12]. The level of enzyme activity has consequences for the tolerability of some anti-malarial drugs; 8-aminoquinolines can cause haemolytic anaemia in G6PD deficient individuals [13, 14].

Polymorphisms in the *HBB* gene, haemoglobin S (HbS, sickle cell trait) and haemoglobin C (HbC) protect against severe malaria [15]. The SNPs are located on chromosome 11 and result in the substitution of a glutamate residue to a valine or lysine residue in the ß-globin chain, respectively. HbAS heterozygote sickle cell trait carriers have a selective advantage in malaria endemic regions as they are protected against severe malaria syndromes [16]. Homozygous HbSS individuals, however, suffer from a serious blood disorder, sickle cell anaemia. Heterozygous HbAC individuals show some protection against severe malaria whereas homozygotes (HbCC) are strongly protected against malaria and, as opposed to HbSS homozygotes, only show symptoms of mild anaemia [17, 18]. Polymorphisms in the *HBB* gene may also increase the likelihood that malaria-infected individuals infect mosquitoes [18, 19].

Considering the implications conferred by human inherited blood disorders (IBD) on malaria disease risk and transmission potential, operationally attractive genotyping tools are of value. Current approaches typically assess a single IBD and whilst basic size electrophoresis for HbC/HbS is practical, G6PD can require multiple PCR’s and visualizations on agarose gel. The assay described here simultaneously assesses four epidemiologically important human markers: *G6PD* G202A and A376G, HbS and HbC. Outcomes of the MagPlex-TAG™ microsphere assay on samples from malaria-exposed populations in Burkina Faso and in The Gambia are compared with established genotyping assays.

## Methods

### DNA from field samples

Study design and whole blood collection were performed as previously described [20]. Briefly, whole blood was collected from males aged 18–45 years in Banfora (Burkina Faso) from August 2014 to November 2015 and from males aged over 10 years in Basse Santa Su, Upper River Region, The Gambia from December 2015 to April 2016. All participants were screened for glucose-6-phosphate enzyme activity using CareStart™ G6PD rapid diagnostic test (G6PD RDT; Access Bio, Inc. Somerset, USA) and the Fluorescent Spot Test (FST) [21]. Both studies were conducted to investigate the safety of primaquine hence only males who had the G6PD enzyme activity measured by point-of-care tests were recruited. A small subset of samples (n = 6) from female participants was made available from another study in Burkina Faso [22] to test the assay on females.

DNA was extracted from 50 µl whole blood from Burkina Faso using the Total NA High Performance kit on a MagNA Pure LC instrument (Roche, Switzerland) and eluted in 50 µl. DNA from The Gambia was extracted from 100 µl cryopreserved blood samples using the DNA Blood Mini kit (Qiagen, Germany) and eluted in 100 µl.

### Assay optimizations

The assay required optimization at the PCR level, the allele specific primer extension (ASPE) level and the median fluorescence intensity (MFI) acquisition level. At the PCR level, the primer concentration (100–300 nM each), the annealing temperature (52.6–65.0 °C) and the template input volume (1–5 µl) were adjusted. At the ASPE level, annealing temperature (51.0 – 57.5 °C) and template volume (1–7 µl) were adjusted in addition to the Biotin-14-dCTP concentration (5–15 µM). At the MFI acquisition level the volume of ASPE product hybridized to beads (1–5 µl) and the concentration of SAPE conjugate (2.5–10 µg/ml) were adjusted. Bovine serum albumin (0.1%, BSA) was added before reading the plate on the MagPix to decrease buffer background MFI levels (Additional file 1: Table S1).

### Genomic PCR amplification

**Fig. 1:**
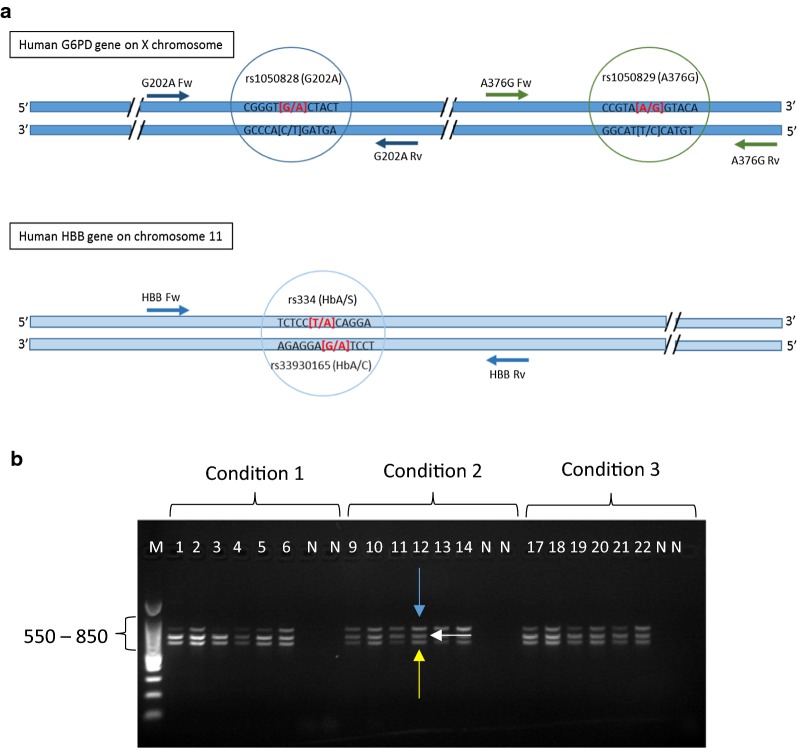
Genomic location and amplification of SNP targets. **a** Top: Representation of the multi-exon *G6PD* gene on the X chromosome. The G202A and the A376G markers are amplified by the primer pairs G202A Fw (forward)/G202A Rv (reverse) and A376G Fw (forward) and A376G Rv (reverse). The G202A allele specific primer extension (ASPE) probes (G202 and A202) anneal to the sense strand and either have a G or an A at their 3′ end (see red highlighted letters in brackets) and the A376G probes (A376 and G376) anneal to the sense strand and either have an A or a G at their 3′ end (see red highlighted letters in brackets). Bottom: Representation of the multi-exon *HBB* gene on chromosome 11. The HbS and HbC markers are amplified by the *HBB* prime pair. The HbS ASPE probes anneal to the sense strand and the HbC ASPE probes anneal to the anti-sense strand (see red highlighted letters in brackets). **b** *G6PD* and *HBB* amplification. Three primer concentrations were tested (conditions 1–3) on human positives (wells 1–22) and negative (N) controls. Condition 1 = equimolar primer concentration (200 nM each), condition 2 = 100 nM each of G202A and A376G primers and 300 nM each of *HBB* primers and condition 3 = 150 nM each of G202A and A376G primers and 300 nM each of *HBB* primers. Even numbered wells correspond to TA = 62.5 °C and odd numbered wells to TA = 60.2 °C. The *HBB* amplicon is 802 bp long (blue arrow), the *G6PD* A376G amplicon is 769 bp long (white arrow) and the *G6PD* G202A amplicon is 619 bp long (yellow arrow)

The gene coding for glucose-6-phosphate dehydrogenase (*G6PD*) is located on the long arm of the X chromosome. The gene contains multiple exons and the SNPs tested (G202A and A376G) are located on exons 5 and 6, respectively. The *HBB* gene is located on the short arm of chromosome 11 and the SNPs of interest (HbS and HbC) are located one base pair apart on exon 1 (Fig. 1a). Three fragments of 619 bp, 769 bp and 802 bp were amplified in the multiplex PCR reaction (Fig. 1b).

Five microliter of extracted DNA was amplified in a 50 µl HotStarTaq Mastermix (Qiagen, Germany) reaction containing 100 nM (each) of *G6PD* primers and 300 nM (each) of *HBB* primers (Table 1). The *G6PD* primers were designed using Primer3 software. For the *HBB* amplification, published primers were used [23]. After amplification, primers and nucleotides from the genomic PCR were removed by adding 3 µl ExoSAP-IT reagent (Applied Biosystems, USA) to 7.5 µl of PCR product.

**Table 1:** Primers and Probes

Genomic PCR primer	
	G202A Fw 5′GTGACCTGGCCAAGAAGAAG3′
G6PD gene	G202A Rv 5′AGGGAGGGAGGCCAAAG3′
	A376G Fw 5′ACACACGGACTCAAAGAGAGG3′
	A376G Rv 5′GGGTCTGAGTGGCCTGAAG3′
HBB gene	*HBB* Fw 5′GAGATATATCTTAGAGGGAGGGC3′
	*HBB* Rv 5′TTTCCCATTCTAAACTGTACCCTGT3′

ASPE probes	Sequence	Bead set
202G	5′**CTAAATCACATACTTAACAACAAA**CCGAAAACACCTTCATCG3′	MTAG-A063
202A	5′**ATACTTTACAAACAAATAACACAC**CCGAAAACACCTTCATCA3′	MTAG-A019
376A	5′**TTAACAACTTATACAAACACAAAC**GCCTCAACAGCCACATGA3′	MTAG-A053
376G	5′**ATCTCAATTACAATAACACACAAA**GCCTCAACAGCCACATGG3′	MTAG-A067
HbA (C)	5′**CTTAAACTCTACTTACTTCTAATT**CATGGTGCATCTGACTCCTG3′	MTAG-A056
HbC	5′**AACTTTCTCTCTCTATTCTTATTT**CATGGTGCATCTGACTCCTA3′	MTAG-A043
HbA (S)	5′**CTAAACATACAAATACACATTTCA**CAGTAACGGCAGACTTCTCCT3′	MTAG-A062
HbS	5′**AATCAACACACAATAACATTCATA**CAGTAACGGCAGACTTCTCCA3′	MTAG-A048

PCR primer sequences and allele specific primer extension (ASPE) probe details. The top of the table contains the primers for genomic PCR amplification and the bottom part of the table contains the ASPE probes. The probe sequence that is complimentary to the anti-tag sequence coupled to the magnetic beads is highlighted in bold. The bead sets were chosen according to the Tag-It^®^ Oligo Design Software v.3.00 (courtesy of Luminex corp., USA)

### Allele specific primer extension (ASPE)

Five microliter of cleaned PCR product was added to 20 µl ASPE reaction mix containing 250 nM (each) of ASPE probes and 200 µM Biotin-14-dCTP (Invitrogen, UK). The ASPE probes were designed using Primer3 software and included the allele specific sequence at their 3′ end. The tag sequences and magnetic beads were chosen according to the manufacturer’s recommendations (Table 1).

### Hybridization and MagPix microsphere multiplex assay

Five µl ASPE reaction was hybridized to 2500 beads of each set, washed twice in 1 × Tm buffer (0.2 M NaCl, 0.1 M Tris, 0.8% TritonX-100, pH 8.0) and incubated for 15 min at 37 °C in 1 × Tm containing 0.1% bovine serum albumin (BSA) and 2.5 µg/ml Streptavidin, R-Phycoerythrin Conjugate (SAPE, Invitrogen, UK). Fifty µl of the reaction was read at 37 °C on a MagPix (Luminex corp., USA). The *G6PD* and *HBB* genotypes were called according to net MFI of the wild type allele compared to the mutated allele (Additional file 1: Table S3A and B). All field samples were repeated at least once. The assay was optimized on positive control DNA to maximize the fluorescent signal and minimize background.

### Flow cytometry for percentage (%) G6PD positive cells

In The Gambia, red blood cells (RBC) were cryopreserved using established protocols [24, 25] and stored at − 80 °C (n = 61 samples, G6PD status confirmed by CareStart™ G6PD RDT, Additional file 2: Table S7). The samples were thawed and re-suspended in equal volume additive (2.5% glucose, 0.9% sodium chloride, 0.027% adenine, 0.75% mannitol) before measuring the % G6PD positive cells by a variation of the methaemoglobin test described in detail in [24, 26]. Samples were examined on a FACScailbur cytometer (Benson & Dickenson) (no gating, 10,000 events, in the FL1 channel 533 + − 30 nm). Analysis was conducted on FlowJo 10.1, and the SED (Super-Enhanced Dmax Subtraction Algorithm, FlowJo 10.1, USA) was calculated. RBCs from anonymous blood donors from the London School of Hygiene and Tropical Medicine were used as controls.

## Results

### Multiplex *HBB* and *G6PD* genotyping on field samples

**Fig. 2:**
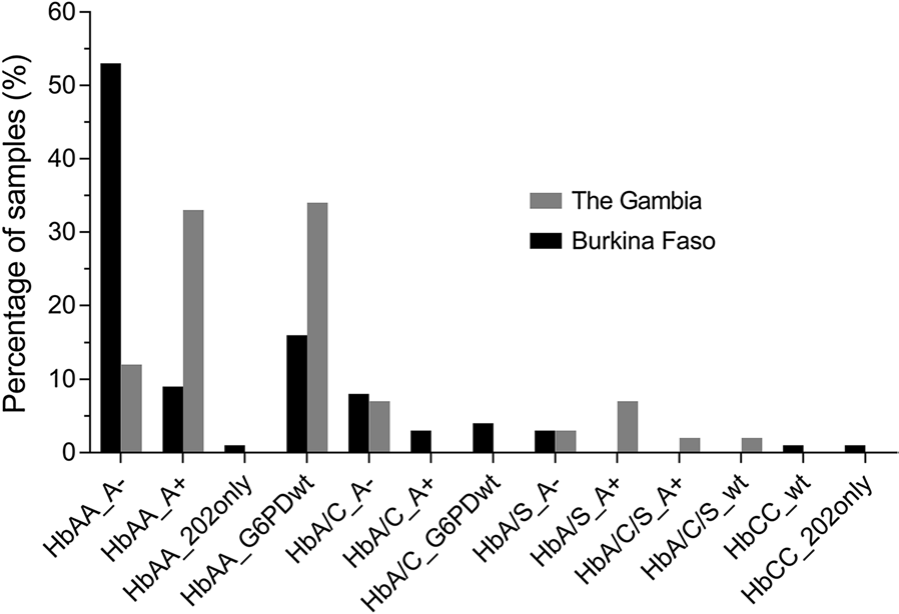
Summary of positive genotyping results of samples from Burkina Faso (n = 75) and The Gambia (n = 58). *G6PD* genotypes correspond to the 202A and 376G alleles (A−), the 202G and 376G alleles (A+), the 202A and 376A alleles (202 only) and the 202G and 376A alleles (G6PDwt, wild type). The *HBB* genotypes are as follows, HbA corresponds to wild type homozygous, HbA/C corresponds to heterozygous A and C alleles, HbA/S corresponds to heterozygous A and S alleles, HbA/C/S corresponds to heterozygous A, C and S alleles and HbCC corresponds to homozygous CC alleles. No homozygous HbSS samples were detected in this study

Seventy-five samples from Burkina Faso (n = 75/78, 96.2%) and fifty-eight samples from The Gambia (n = 58/61, 95.1%) had a *G6PD* and a *HBB* genotype successfully assigned by the bead-based assay (Fig. 2). Three samples from Burkina Faso and two samples from The Gambia were excluded due to net MFI values below the arbitrary cut-off of mean MFI plus two standard deviations (mean MFI + 2SD) (Additional file 1: Table S2). This cut-off was chosen to unambiguously separate positive fluorescence signal from background signal. One sample from The Gambia failed to amplify in the initial PCR reaction. The *G6PD* and *HBB* genotypes of the remaining samples were called according to net MFI of the wild type allele compared to the mutated allele (Additional file 1: Table S3A and B). In Burkina Faso, the majority of samples (53.3%, 40/75) were *HBB* wild type and contained both the 202A and 376G SNPs (A−). Two samples contained the 202A (mutation) SNP in the absence of the 376G (mutation) SNP. HbAS was detected in 2.7% (2/75) and HbAC in 14.7% (11/75) of samples. Two individuals were homozygous HbCC.

In The Gambia, 32.8% (19/58) were *HBB* wild type and 202G (wild type) and 376G (mutation, A+); HbAS was detected in 13.8% (8/58) and HbAC in 10.3% (6/58) of samples. Two individuals were heterozygous carriers for both the HbC and HbS alleles. Homozygous HbSS was not detected in either population.

To determine assay performance in *G6PD* normal or heterozygous female individuals, a small subset of samples (n = 6) from a separate study conducted in Burkina Faso [22] were tested. *G6PD* SNPs were successfully genotyped in all samples irrespective of gender (Additional file 1: Table S4).

### *G6PD* genotyping method comparison

The genotyping data generated here was compared to TaqMan OpenArray genotyping data [13] and KASP chemistry data [27] available for all samples (Burkina Faso, n = 78 and The Gambia, n = 61). Eight samples failed in the KASP assay and three samples failed in the TaqMan assay. The allele calls for all available samples agreed by all assays, only a single KASP genotyping call disagreed (data available for 139 sample pairs, Additional file 2: Table S6).

### *G6PD* genotype and phenotype comparison

Sixty-one cryopreserved red blood samples from The Gambia (males only) were analysed by flow cytometry for percentage (%) G6PD positive cells, used here as a proxy of G6PD enzyme activity (Additional file 2: Table S7). There were no indications of haemolysis in samples upon visual inspection. For four samples (4/61) the % of G6PD positive cells was below that of the negative controls and was set to zero. Flow cytometry results were stratified by G6PD genotype (Additional file 1: Figure S2). G6PD wild type samples (202G and 376A; n = 23) had the greatest % G6PD positive cells (median 89.5%, IQR, 77.1–93.7); deficient A− samples (202A and 376G; n = 13) displayed the lowest % G6PD positive cells (median 2.37%, IQR 0.9–16.7). The A+ samples (202A and 376G; n = 25, median G6PD positive cells 55.3%, IQR 4.81–79.4) contained two populations of cells: one similar to wild type samples (n = 17, median 72.3%, IQR 55.3–86.3) and the other similar to A− samples (n = 8, median 1.51%, IQR 0.005–3.84). Interestingly, outliers were detected in the wild type populations and in the A− populations (Additional file 1: Figure S2). Three out of four wild type samples (0%, 22.5% and 40.3% G6PD positive cells respectively) with lower than expected % G6PD positive cells tested deficient by CareStart G6PD RDT. The fourth wild type outlier contained 26.0% G6PD positive cells and tested normal by CareStart G6PD RDT. The four A− samples with higher than expected % G6PD positive cells contained 16.7%, 29.3%, 40.5% and 77.1% G6PD positive cells respectively and all four samples tested deficient by CareStart G6PD RDT (Additional file 2: Table S7).

**Fig. 3:**
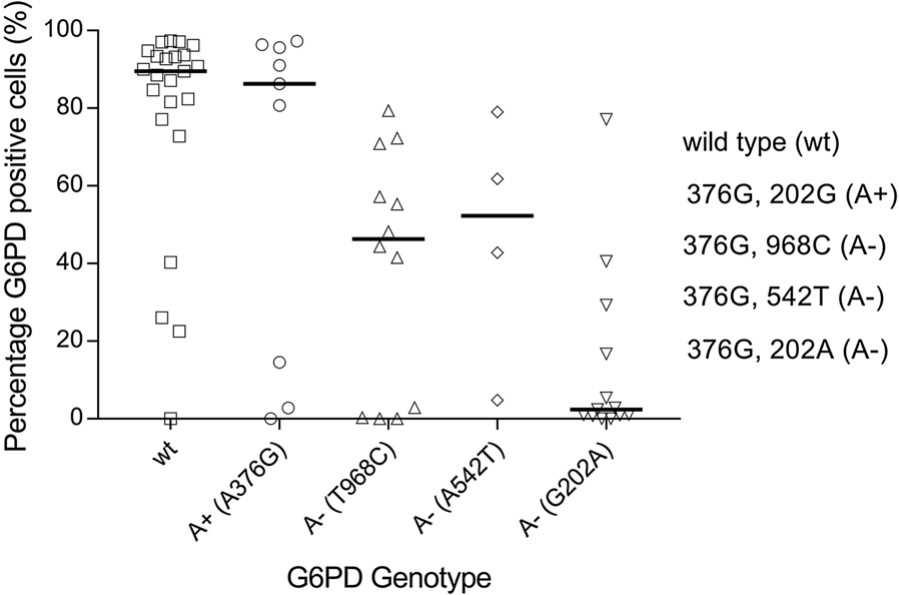
Percentage G6PD positive cells by flow cytometry by *G6PD* genotype. The % G6PD positive cells assessed by FACS was plotted against *G6PD* genotype generated by magnetic bead-based multiplex assay and KASP assay; wild type (wt, 202G and 376A), A+ (202G and 276G), A− (202A and 376G), A− (202A and 542TG) and A− (202A and 968C). The black horizontal line represents the median. The % G6PD positive cells were calculated using FlowJo version 10 Super-Enhanced Dmax Subtraction (SED) algorithm

Genotyping data generated by PCR-based KASP chemistry was available for all samples with cryopreserved RBC data (n = 61, Sepulveda and Grignard, pers. comm., and [27]) and contained the two additional alleles commonly found in The Gambia; the Betica-Selma variant (T968C) and the Santamaria variant (A542T). The two alleles were only present in samples that also had the 376G mutation and the samples were hence classed as A− genotypes. The 376G/968C (n = 12) and the 376G/542T (n = 4) mutations had a median % G6PD positive cells of 46.3% (IQR 1.5–64.0) and 52.3% (IQR 23.8–70.4), respectively. Only nine samples were A+ (376G only) and these had a median % G6PD positive cells of 86.3 (IQR 14.5–95.6). Now, only three A+ genotypes had low % G6PD positive cells, suggesting that some of the outliers in this group were explained by alternative SNPs (968C and 542T) affecting G6PD activity (Fig. 3).

### Sample analysis and cost

Using the microsphere assay for the four markers, data for 96 samples was available within 7 h at an estimated cost per sample of $ 4.30 (calculated December 2017, Additional file 1: Table S5). Standard protocols for *G6PD* and *HBB* genotyping involve a PCR reaction followed either by a restriction digestion (PCR–RFLP) or Sanger sequencing [23, 28]. The cost per sample for the four markers using standard protocols was estimated at $ 5.84 per sample with 40 working hours processing time.

## Discussion

This study describes a method for simultaneous assessment of G6PD-deficiency markers and *HBB* polymorphisms. These were successfully genotyped from small volumes (5 µl) of DNA extracted from human blood. Gene segments were amplified in a single, multiplex PCR reaction and genotyped by magnetic bead-based allele specific multiplex assay. The assay is fast, relatively inexpensive and performed as well as established genotyping methods.

Assessing G6PD deficiency status is relevant to predict the susceptibility of individuals for the disadvantageous haemolytic consequences of treatment with 8-aminoquinolines like primaquine and tafenoquine [29–33]. Although G6PD phenotyping assays [21, 34, 35] may be most informative in this respect and allow meaningful assessments of varying levels of G6PD due to random X-inactivation or lyonization [24–26, 36, 37], genotyping approaches are frequently used in population research to plan public health interventions [11, 38]. Here, a multiplex assay that concurrently detects common SNPs for the A− *G6PD* variant and *HBB* mutations was successfully developed using a mix of previously published and novel oligonucleotides [23] and optimized to maximize the SNP specific fluorescence signal. Using this microsphere multiplex assay, samples from G6PD deficient and G6PD normal individuals were assessed for mutations with good agreement with conventional genotyping methods. To assess the correlation in between *G6PD* genotype and G6PD enzyme activity, the % G6PD-positive cells was assessed for different *G6PD* genotypes. In line with published data, the A− genotype (376G and 202A) had the lowest percentage G6PD positive cells compared to wild type and A+ individuals [39]. A small proportion of genetically deficient individuals did not have a lower percentage G6PD positive cells and, conversely, a fraction of genetically *G6PD* normal individuals displayed unexpected low percentage G6PD positive cells. Including additional SNPs that have been associated with lower G6PD activity in The Gambia [8], improved the correlation between percentage G6PD positive cells and genotypes. This suggests that genotyping approaches that focus only on 376G and 202A may misclassify individuals with low G6PD enzyme activity. The inability to explain all variation in G6PD activity by the examined SNPs suggests that other (currently unidentified) genetic variants that affect G6PD status may exist.

The current genotyping results were based on populations enriched for G6PD deficient individuals and findings thus do not represent G6PD deficiency prevalence in the two countries. Whilst the current selective sample set does not allow unbiased population prevalence estimates, the HbS gene was more common in The Gambia than Burkina Faso (13.8% vs. 2.7%) and the HbC trait was more common in Burkina Faso (14.7% vs. 10.3%) [16, 40].

The assay presented here has an advantage that it can be adapted to multiple SNPs of interest and is increasingly used for detecting SNPs associated with drug resistance in parasites [41, 42]. The assay does not detect large deletions such as either of the thalassaemias (α-thalassaemia and β-thalassaemia), which is one of its limitations. At present the assay contains two common *G6PD* SNPs and two *HBB* gene polymorphisms, but the flexible multiplex nature of the assay allows users to include additional markers. The assay is less time consuming and labour intensive than traditional end-point PCR/agarose gel genotyping methods and requires less operator time. Up to 50 markers can be tested in a single reaction and markers can be mixed and matched according to population of interest and parasite epidemiology.

## Conclusions

The microsphere multiplex assay presented in the current study may play a role in addressing the increasing need to test for human and parasite genetic changes because of their impact on disease progression and malaria epidemiology. The multiplex nature of the assay in addition to the fast turn-around time and the relatively low cost, make it attractive for assessment of multiple genetic markers in large-scale epidemiological studies.

## Additional files

**Additional file 1: Table S1.** Optimisation flow chart: A. Genomic PCR, B. ASPE (allele specific primer extension) and C. Hybridisation of ASPE reactions to microspheres. Optimal conditions in black. **Table S2.** Cut-off MFI values for each marker. The mean plus two standard deviations (Mean + 2SD) of the background MFI signal of each marker was chosen as a cut-off value. An MFI signal greater than the cut-off scored a positive result. **Table S3.** Examples of how the net median fluorescence intensity (MFI) is recorded for each allele and the percentage wild type MFI (WT %) is calculated. A. For G6PD (G202A and A376G) in males (males only have a single copy of the G6PD gene), above 75% wild type MFI signifies that the sample is wild type and below 25% wild type MFI signifies that the gene carries a mutation. In males, if the percentage wild type MFI is between 25%-75%, the genotyping needs to be repeated. In HBB (HbC and HbS), above 75% wild type MFI signifies homozygous wild type and below 25% wild type MFI signifies a mutation on both alleles. A percentage wild type MFI and a percentage mutated MFI in between 25% and 75% signifies that the sample is heterozygous and contains both a wild type and mutated allele. The G6PD genotypes are as follows: A- samples carry both the 202A and 376G mutations and the A+ samples carry the 202G wild type allele and the 376G mutation. B. The HBB genotypes can be either homozygous HbAA, HbCC or HbSS. Heterozygous genotypes contain a combination of genotyping calls, for example, HbA/C samples are heterozygous samples with a wild type allele (HbA) and a mutated HbC allele. This sample does not contain the HbS mutation on either of its two alleles. An HbA/C/S genotype is heterozygous for the both HbC and HbS alleles. Samples with net MFI values below the mean MFI plus two standard deviations (Mean + 2SD) for that marker are highlighted with a * (see Table S2). These samples were excluded from further analysis. **Table S4.** G6PD and HBB genotyping on samples from female participants. Six samples from females were analysed to show that the assay is working for homozygous and heterozygous women. **Table S5.** Pricing and time. **Figure S1.** Example of comparison of G6PD activity in normal (left) and deficient (right) individuals. The grey-shaded peaks correspond to samples and the blue-outlined peaks correspond to untreated negative controls. The log-10 fluorescence in the FL1 channel (533 ± 30 nm) is measured (x-axis). The SE Dmax % positive and the Overton % positive detect the % of positive cells against the control. The SE Dmax additionally accounts for outliers. **Figure S2.** Flow cytometry results by G6PD genotype. The percentage of G6PD positive cells was plotted by G6PD genotype generated by magnetic bead-based multiplex assay; wild type (wt, 202G and 376A), A+ (202G and 276G) and A- (202A and 376G). The % G6PD positive cells were calculated using FlowJo version 10 Super-Enhanced Dmax Subtraction (SED) algorithm.

**Additional file 2: Table S6.** G6PD G202A & A376G genotyping comparison. The MagPix genotyping calls were compared to data generated by TaqMan OpenArray and KASP PCR-based genotyping. Wild type calls are 202G and 376A, A- calls are 202A and 376G and A+ calls are 202G and 376G. A dot (.) denotes samples for which genotyping was unsuccessful and samples marked with a * had low net MFI values (net MFI < net MFI + 2SD). **Table S7.** G6PD percentage positive cells (G6PD % positive), genotyping amd CareStart G6PD RDT results for all samples from The Gambia. Samples were initially stratified by RDT result. The KASP genotypes include additional SNPs, T968C and A542T, that have been associated with decreased G6PD enzyme activity in The Gambia. The samples containing the mutant alleles are highlighted in green and the % G6PD positive cells are marked in green. Samples labelled with a * did not have a MagPix genotype assigned and hence had their KASP G6PD (G202A and A376G) used in this comparison.

## Abbreviations

G6PDd: glucose-6-phosphate dehydrogenase deficiency
HbC: haemoglobin C
HbS: haemoglobin S
IBD: inherited blood disorders
SNPs: single nucleotide polymorphisms
ASPE: allele specific primer extension
MFI: median fluorescence intensity
G6PD: glucose-6-phosphate dehydrogenase
RDT: rapid diagnostic test
FST: Fluorescent Spot Test
RBC: red blood cell
SED: Super-Enhanced Dmax Subtraction
