# Supplementary material for: Bead-based assays to simultaneously detect multiple human inherited blood disorders associated with malaria
Source: Malar J. 2019 Jan 21;18:14. doi: 10.1186/s12936-019-2648-7 (PMC6341711; doi:10.1186/s12936-019-2648-7)
Supplement: Supplementary file 1 — Additional file 1: Table S1. Optimisation flow chart: A. Genomic PCR, B. ASPE (allele specific primer extension) and C. Hybridisation of ASPE reactions to microspheres. Optimal conditions in black. Table S2. Cut-off MFI values for each marker. The mean plus two standard deviations (Mean + 2SD) of the background MFI signal of each marker was chosen as a cut-off value. An MFI signal greater than the cut-off scored a positive result. Table S3. Examples of how the net median fluorescence intensity (MFI) is recorded for each allele and the percentage wild type MFI (WT %) is calculated. A. For G6PD (G202A and A376G) in males (males only have a single copy of the G6PD gene), above 75% wild type MFI signifies that the sample is wild type and below 25% wild type MFI signifies that the gene carries a mutation. In males, if the percentage wild type MFI is between 25%-75%, the genotyping needs to be repeated. In HBB (HbC and HbS), above 75% wild type MFI signifies homozygous wild type and below 25% wild type MFI signifies a mutation on both alleles. A percentage wild type MFI and a percentage mutated MFI in between 25% and 75% signifies that the sample is heterozygous and contains both a wild type and mutated allele. The G6PD genotypes are as follows: A- samples carry both the 202A and 376G mutations and the A+ samples carry the 202G wild type allele and the 376G mutation. B. The HBB genotypes can be either homozygous HbAA, HbCC or HbSS. Heterozygous genotypes contain a combination of genotyping calls, for example, HbA/C samples are heterozygous samples with a wild type allele (HbA) and a mutated HbC allele. This sample does not contain the HbS mutation on either of its two alleles. An HbA/C/S genotype is heterozygous for the both HbC and HbS alleles. Samples with net MFI values below the mean MFI plus two standard deviations (Mean + 2SD) for that marker are highlighted with a * (see Table S2). These samples were excluded from further analysis. Table S4. G6PD and HBB genotyping [file 12936_2019_2648_MOESM1_ESM.docx]

| **Additional tables and figures** |
| --- |
|  |
| \| A  1.1 Primer concentration  G6PD 202 & 376, 100 nM each; HbC/S 300 nM  **1. Genomic PCR optimisation**  G6PD 202 & 376 & HbC/S, 200 nM each  G6PD 202 & 376, 150 nM each; HbC/S 300 nM  1.2 Annealing T° (65°C-52.6°C)  60.2°C  1.3 Template volume  2.5 µl  5 µl  1 µl \| \| --- \| \| B  2.1 Biotin-14-dCTP concentration  **2. ASPE optimisation**  5 µM & 7.5 µM  2.2 Annealing T° (57.5°C-51.0°C)  53.5°C  2.3 PCR product input volume  7 µl  5 µl  1 µl  10 µM  12.5 µM & 15 µM \| \| C  3.1 ASPE input volume  **3. Hybridisation optimisation**  1 µl  3.2 SAPE concentration  2.5 µg/ml  3.3 0.1% BSA  Yes  No  5 µl  5 µg/ml  10 µg/ml \| |
|  |
|  |
| **Table S1.** Optimisation flow chart: A. Genomic PCR, B. ASPE (allele specific primer extension) and C. Hybridisation of ASPE reactions to microspheres. Optimal conditions in black. |
|  |
|  |

| Marker | Cut-off MFI value (Mean + 2SD of background) |
| --- | --- |
| HbA/C | 58 |
| HbC | 0 |
| HbA/S | 55 |
| HbS | 48 |
| 202G_WT | 89 |
| 202A | 68 |
| 376A_WT | 83 |
| 376G | 118 |

**Table S2.** Cut-off MFI values for each marker. The mean plus two standard deviations (Mean + 2SD) of the background MFI signal of each marker was chosen as a cut-off value. An MFI signal greater than the cut-off scored a positive result.

| 3a | 202G_WT  MFI | 202A  MFI | % 202 WT MFI | 376A_WT  MFI | 376G  MFI | % 376 WT MFI | genotype202 | genotype376 | G6PD type |
| --- | --- | --- | --- | --- | --- | --- | --- | --- | --- |
| Gambia_1* | 219 | 0 | 100 | 0 | 62 | 0 | wt | DEF | A+ |
| Gambia_2 | 419 | 0 | 100 | 0 | 459 | 0 | wt | DEF | A+ |
| Gambia_3 | 400 | 0 | 100 | 491.5 | 0 | 100 | wt | wt | wt |
| Gambia_4 | 186 | 42 | 82 | 47 | 310 | 13 | wt | DEF | A+ |
| Gambia_5 | 236 | 65 | 78 | 487 | 64 | 88 | wt | wt | wt |
| Gambia_6 | 507 | 8 | 98 | 672 | 7 | 99 | wt | wt | wt |

| 3b | HbA/C  MFI | HbC  MFI | % HbA/C WT MFI | HbA/S  MFI | HbS  MFI | % HbA/S WT MFI | genotype HbA/C | genotype HbA/S | HBB type |
| --- | --- | --- | --- | --- | --- | --- | --- | --- | --- |
| Gambia_1 | 897 | 0 | 100 | 707 | 0 | 100 | HbA | HbA | HbAA |
| Gambia_2 | 1019 | 0 | 100 | 865 | 0 | 100 | HbA | HbA | HbAA |
| Gambia_3 | 233 | 502 | 32 | 620 | 278 | 69 | HbA/C | HbA/S | HbA/C/S |
| Gambia_4 | 865 | 80 | 92 | 964 | 72 | 93 | HbA | HbA | HbAA |
| Gambia_5 | 1228 | 80 | 94 | 1243 | 82 | 94 | HbA | HbA | HbAA |
| Gambia_6 | 568 | 20 | 97 | 716 | 25 | 97 | HbA | HbA | HbAA |

**Table S3.** Examples of how the net median fluorescence intensity (MFI) is recorded for each allele and the percentage wild type MFI (WT %) is calculated. A. For G6PD (G202A and A376G) in males (males only have a single copy of the G6PD gene), above 75% wild type MFI signifies that the sample is wild type and below 25% wild type MFI signifies that the gene carries a mutation. In males, if the percentage wild type MFI is between 25%-75%, the genotyping needs to be repeated. In HBB (HbC and HbS), above 75% wild type MFI signifies homozygous wild type and below 25% wild type MFI signifies a mutation on both alleles. A percentage wild type MFI and a percentage mutated MFI in between 25% and 75% signifies that the sample is heterozygous and contains both a wild type and mutated allele. The G6PD genotypes are as follows: A- samples carry both the 202A and 376G mutations and the A+ samples carry the 202G wild type allele and the 376G mutation. B. The HBB genotypes can be either homozygous HbAA, HbCC or HbSS. Heterozygous genotypes contain a combination of genotyping calls, for example, HbA/C samples are heterozygous samples with a wild type allele (HbA) and a mutated HbC allele. This sample does not contain the HbS mutation on either of its two alleles. An HbA/C/S genotype is heterozygous for the both HbC and HbS alleles. Samples with net MFI values below the mean MFI plus two standard deviations (Mean + 2SD) for that marker are highlighted with a * (see Table S2). These samples were excluded from further analysis.

| ID/G6PD | 202G_WT  MFI | 202A  MFI | % 202 WT MFI | 376A_WT  MFI | 376G  MFI | % 376 WT MFI | genotype202 | genotype376 | G6PD type |
| --- | --- | --- | --- | --- | --- | --- | --- | --- | --- |
| Sample 1 | 517 | 41.75 | 93 | 33.5 | 573 | 6 | wt | DEF | A+ |
| Sample 2 | 303 | 18.75 | 94 | 131.5 | 86 | 60 | wt | het | het376 |
|  |  |  |  |  |  |  |  |  |  |
| Sample 3 | 298 | 504.75 | 37 | 424.5 | 357 | 54 | het | het | het |
| Sample 4 | 292 | 498.75 | 37 | 492.5 | 419 | 54 | het | het | het |
|  |  |  |  |  |  |  |  |  |  |
| Sample 5 | 437 | 53.75 | 89 | 594.5 | 58 | 91 | wt | wt | wt |
| Sample 6 | 40 | 568 | 7 | 46 | 361 | 11 | DEF | DEF | A- |

| ID/HBB | HbA/C  MFI | HbC  MFI | % HbA/C WT MFI | HbA/S  MFI | HbS  MFI | % HbA/S WT MFI | genotype HbA/C | genotype HbA/S | HBB type |
| --- | --- | --- | --- | --- | --- | --- | --- | --- | --- |
| Sample 1 | 827 | 933 | 47 | 1253 | 35 | 97 | HbA/C | HbAA | HbA/C |
| Sample 2 | 469 | 22 | 96 | 367 | 13 | 97 | HbAA | HbAA | HbAA |
|  |  |  |  |  |  |  |  |  |  |
| Sample 3 | 791 | 19 | 98 | 753 | 19 | 98 | HbAA | HbAA | HbAA |
| Sample 4 | 1066 | 42 | 96 | 646 | 1193 | 35 | HbAA | HbA/S | HbA/S |
|  |  |  |  |  |  |  |  |  |  |
| Sample 5 | 1239 | 48 | 96 | 1157 | 46 | 96 | HbAA | HbAA | HbAA |
| Sample 6 | 813 | 42 | 95 | 728 | 42 | 95 | HbAA | HbAA | HbAA |

**Table S4.** G6PD and HBB genotyping on samples from female participants. Six samples from females were analysed to show that the assay is working for homozygous and heterozygous women.

**Table S5.** Pricing and time

|  | Multiplex bead assay | Standard protocol** |
| --- | --- | --- |
| Price ($)/reaction* | 4.30 | 5.84 |
| Price ($)/96 samples | 412.80 | 560.64 |
| Time (hours)*** | 6.45 | 40 |

*Price per reaction from extracted DNA

** Assuming 4 PCRs necessary to interrogate SNPs G6PD 202, G6PD 376, HbC and HbS.

***Working hours

**Figure S1.** Example of comparison of G6PD activity in normal (left) and deficient (right) individuals. The grey-shaded peaks correspond to samples and the blue-outlined peaks correspond to untreated negative controls. The log-10 fluorescence in the FL1 channel (533 ± 30 nm) is measured (x-axis). The SE Dymax % positive and the Overton % positive detect the % of positive cells against the control. The SE Dymax additionally accounts for outliers.

**Figure S2.** Flow cytometry results by G6PD genotype. The percentage of G6PD positive cells was plotted by G6PD genotype generated by magnetic bead-based multiplex assay; wild type (wt, 202G and 376A), A+ (202G and 276G) and A- (202A and 376G). The % G6PD positive cells were calculated using FlowJo version 10 Super-Enhanced Dmax Subtraction (SED) algorithm.
